# Controlling the optical and structural properties of ZnS–AgInS_2_ nanocrystals by using a photo-induced process

**DOI:** 10.3762/bjnano.5.187

**Published:** 2014-10-14

**Authors:** Takashi Yatsui, Fumihiro Morigaki, Tadashi Kawazoe

**Affiliations:** 1 School of Engineering, University of Tokyo, Bunkyo-ku, Tokyo 113-8656, Japan

**Keywords:** low toxicity, self-assembly, visible-light-emitting nanocrystals, ZnS–AgInS_2_ (ZAIS)

## Abstract

ZnS–AgInS_2_ (ZAIS) solid-solution nanocrystals are promising materials for nanophotonic devices in the visible region because of their low toxicity and good emission properties. We developed a technique of photo-induced synthesis to control the size and composition of the ZAIS nanocrystals. This method successfully decreased the defect levels, as well as the size and size variation of ZAIS nanocrystals by controlling the excitation wavelength during synthesis. Detailed analysis of transmission electron microscope images confirmed that the photo-induced synthesis yielded a high crystallinity of the ZAIS nanocrystals with small variations in size and content.

## Introduction

Continued innovation in optical technology is essential for the advancement of information-processing systems. In particular, it is important to reduce both the size and energy consumption of photonic devices to ensure successful integration [1]. To reduce the size of photonic devices beyond the diffraction limit, the photons should be coupled with material excitation such as electrons in metallic materials [2]. To avoid absorption loss in metallic materials, we have proposed nanophotonic devices using semiconductor quantum structures, including quantum cubes [3], quantum dots (QDs) [4], quantum wells [5], and quantum rings [6]. Kawazoe et al., have demonstrated the room-temperature operation of AND-gate and NOT-gate devices using InAs QD pairs [7]. In a nanophotonic device, near-field energy-transfer via a dipole-forbidden energy state, which is unattainable in conventional photonic devices, is used [8]. The near-field energy-transfer originates from an exchange of virtual photons between the resonant energy states [9], where the virtual photons on a nanoparticle activate the dipole-forbidden energy state.

The successful fabrication of a nanophotonic device requires the control of its size to ensure that the quantized energy levels are resonant to facilitate an efficient optical near-field interaction. The solution process could be a promising process for this purpose because it can easily regulate the size and shape by controlling the growth kinetics [10]. It affords precise control over the size of CdSe QDs with a very low size variation that is as small as a fifth of the atomic interface [11], and it has been used to manufacture commercially available display devices [12]. On the other hand, the reduction of toxic components such as Cd and Se is required. Therefore, ZnS–AgInS_2_ solid-solution (ZAIS) nanocrystals [13] are promising materials for nanophotonic devices in the visible region because of their low toxicity. In addition, since ZAIS nanocrystals have long decay times for emissions [14], it can be applied to optical buffer memory [15]. To realize a room-temperature operation of nanophotonic devices, the spectrum width of photoluminescence (PL) needs to be narrowed. Emission-wavelength controlled nanocrystals with a narrow spectral range are also of great interest for display devices. This is because nanometer-scale control in wavelength is required for the color display to tune the color rendering index. To meet this requirement, size control at the scale of single atoms is required [12]. We performed laser-assisted synthesis of ZAIS nanocrystals to meet those requirements.

## Results and Discussion

### Photo-induced synthesis

**Figure 1:**
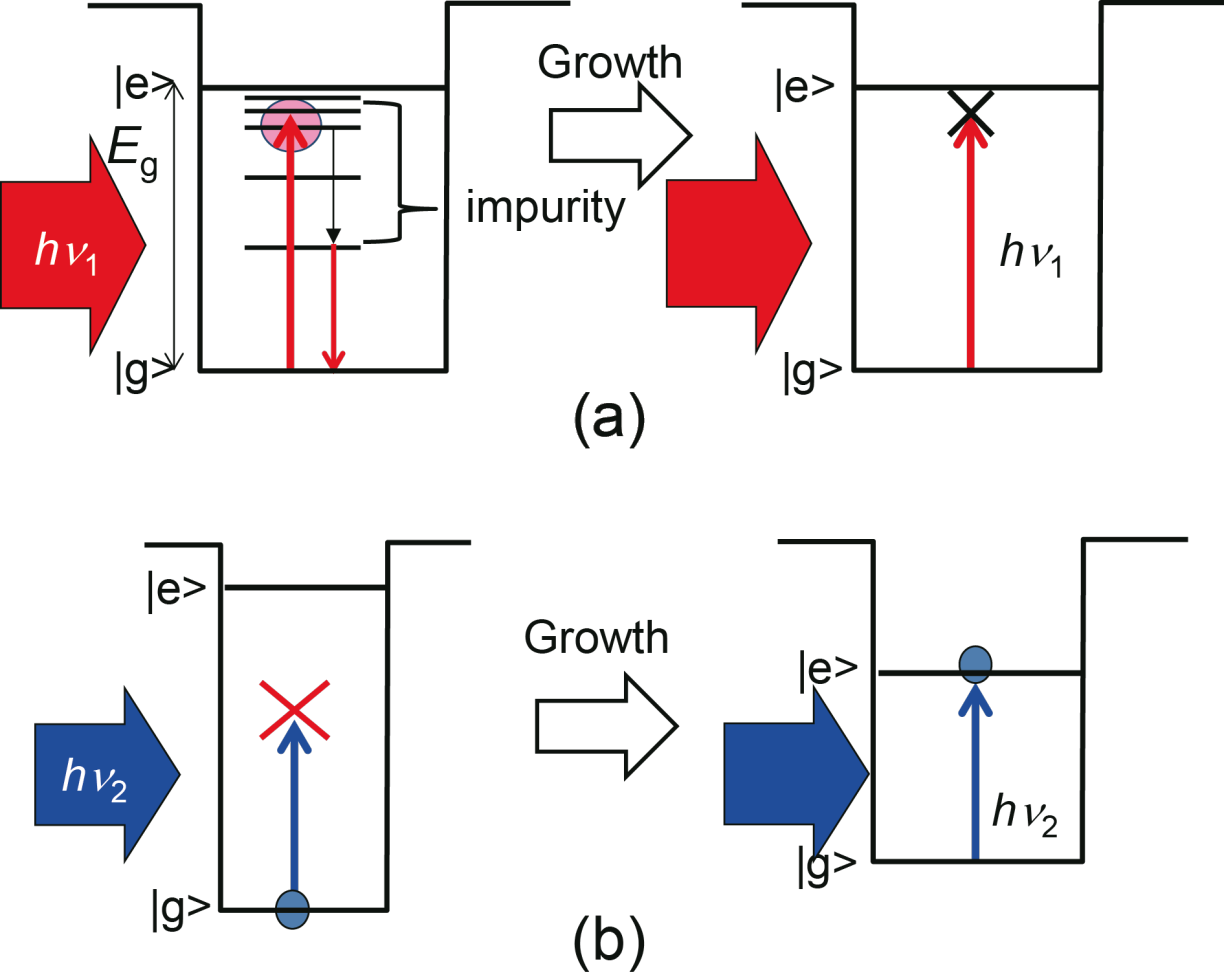
Schematic of the photo-induced synthesis. (a) Selective etching of defect levels. The relationship between the energy of an incident photon and the band structure of a ZAIS nanocrystal is shown in the left panel. As growth proceeds, the defect levels disappear, resulting in the high-quality nanocrystal at the end (right panel). (b) Controlling the size of a nanocrystal. The relationship between the energy of an incident photon and the band structure of a ZAIS nanocrystal is shown in the left panel. As growth proceeds, the size of the nanocrystal increases and the band gap energy decreases below the energy of the incident photon. Electron–hole pairs are then excited and trigger an oxidation–reduction reaction in the ZAIS nanocrystal and growth halts (right panel).

Defects or impurities must be removed to reduce the spectral width and to obtain a higher crystal quality [14,16]. Since the energy level corresponding to a defect or an impurity in ZAIS nanocrystals is lower than the band gap energy (*E*_g_), the excited carriers are trapped at the inter-band defect levels. Consequently, the quantum efficiency of the ZAIS nanocrystals decreases if a larger number of defect levels is present. However, when illuminated by a photon with an energy of *h*ν_1_, which is larger than the defect levels and smaller than *E*_g_, defect levels are removed preferentially. This occurs because photo-induced etching takes place in the areas with defects as a result of local oxidation–reduction reactions after the excited electron–hole pairs have relaxed to those defect areas in ZAIS nanocrystals (Figure 1a). During the photo-synthesis of ZAIS nanocrystals with the illumination photon energies exceeding *E*_g_, excitons induce an oxidation–reduction reaction in the nanocrystals. Consequently, the etching of the deposited ZAIS atoms on the nanocrystals surface proceeds. The growth rate is controlled by the absorbed light intensity and wavelength, which control the nanocrystal size. Similar photo-synthesis for controlling the size of nanocrystals have been reported for CdSe [14], ZnO [16–17], and Si [18] (Figure 1b).

We measured the excitation spectra of the synthesized ZAIS nanocrystals to find the optimum wavelength for controlling the spectra. Based on the synthesis method described in [13], solid-solution nanocrystals of ZAIS were synthesized by thermal decomposition of a metal-ion–diethyldithiocarbamate complex of (AgIn)*_x_*Zn_2(1−_*_x_*_)_(S_2_CN(C_2_H_6_)_2_)_4_. Here, we set *x* to 0.5 for all experiments. By using 50 mg of the precursor powder, ZAIS nanocrystals were synthesized as follows: Step (1): The precursor was annealed at 180 °C for 30 min in a N_2_ atmosphere, yielding a brown powder. Step (2): Oleylamine was added to the brown powder obtained in step (1), followed by further annealing at 180 °C for a time *t* (defined as the growth time for this process) in a N_2_ atmosphere to grow ZAIS nanocrystals. During the crystal growth in this annealing process, irradiation with light was introduced to control the size and crystallinity. Step (3): Large particles were removed from the resulting suspension by centrifugation. By adding methanol, the ZAIS nanocrystals were separated from the supernatant.

**Figure 2:**
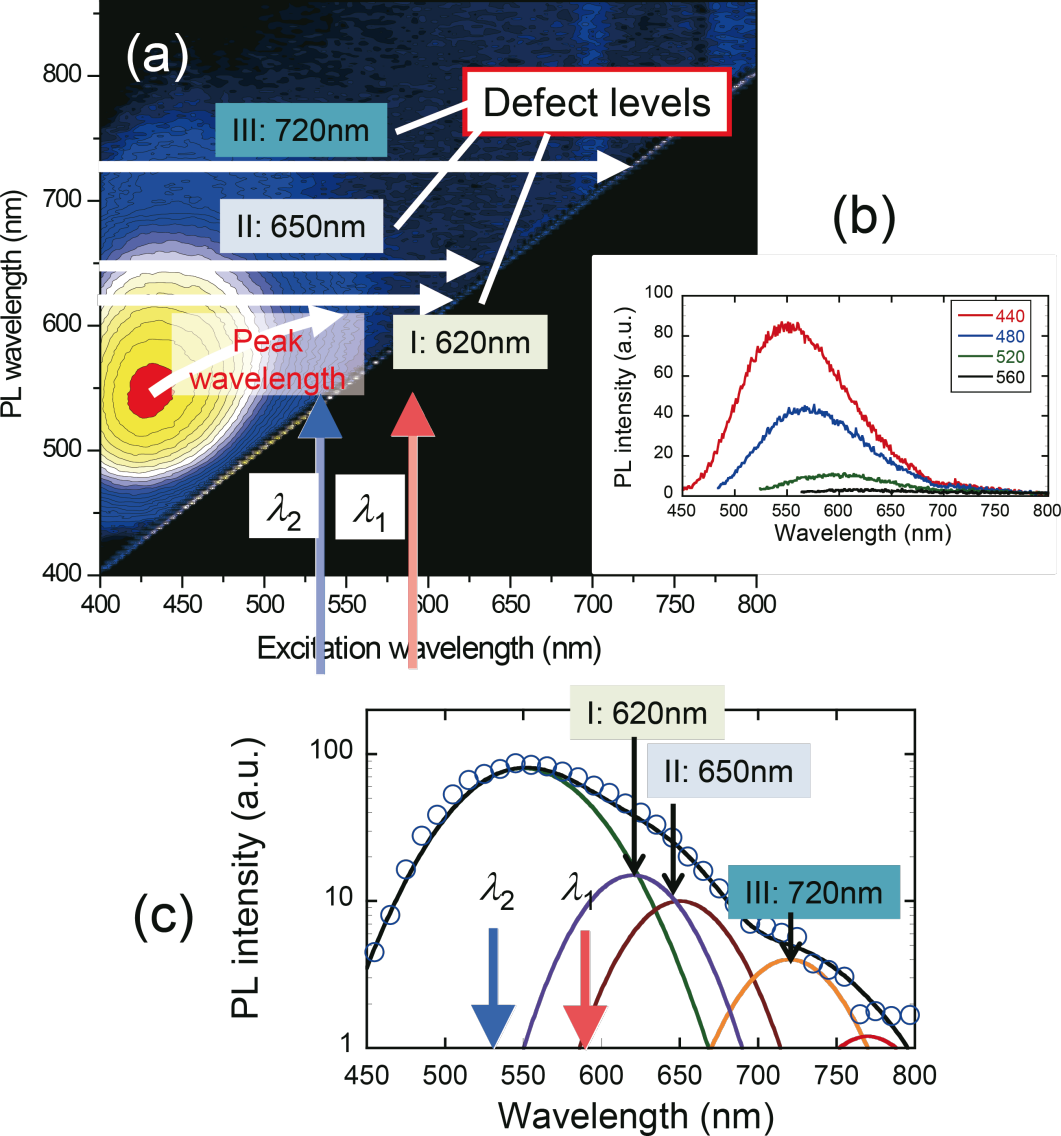
(a) Dependence of PL spectra on excitation wavelength. (b) PL spectra obtained with excitation wavelengths of 440, 480, 520, and 560 nm. (c) Fitted PL spectrum (the same as the red curve in (b)) and several Lorenz curves with peak wavelengths of 550, 620, 650, and 720 nm.

Figure 2a and Figure 2b show the excitation spectra of fabricated ZAIS without laser irradiation during step (2), in which ZAIS nanocrystals were synthesized in *t* = 60 min. Figure 2c shows a typical emission spectrum obtained after excitation at 440 nm. This spectrum had a broad spectral width, with several emission peaks at 620 (peak I), 650 (peak II), and 720 nm (peak III) in addition to the main emission peak at around 550 nm. The main peak should have originated from the emission from the band edge of ZAIS and the remaining peaks should have originated from the defect levels. Based on these results, 593 nm light (λ_1_) was chosen to decrease the number of impurity sites. As this wavelength was longer than the spectral peak wavelength around 550 nm, it prevented carrier excitation in the ZAIS nanocrystals (see Figure 1a). In addition, to control the size of the ZAIS nanocrystals, 532 nm light (λ_2_) was used so that the ZAIS nanocrystals would absorb the light (see Figure 1b).

### Selective reduction of defect levels

**Figure 3:**
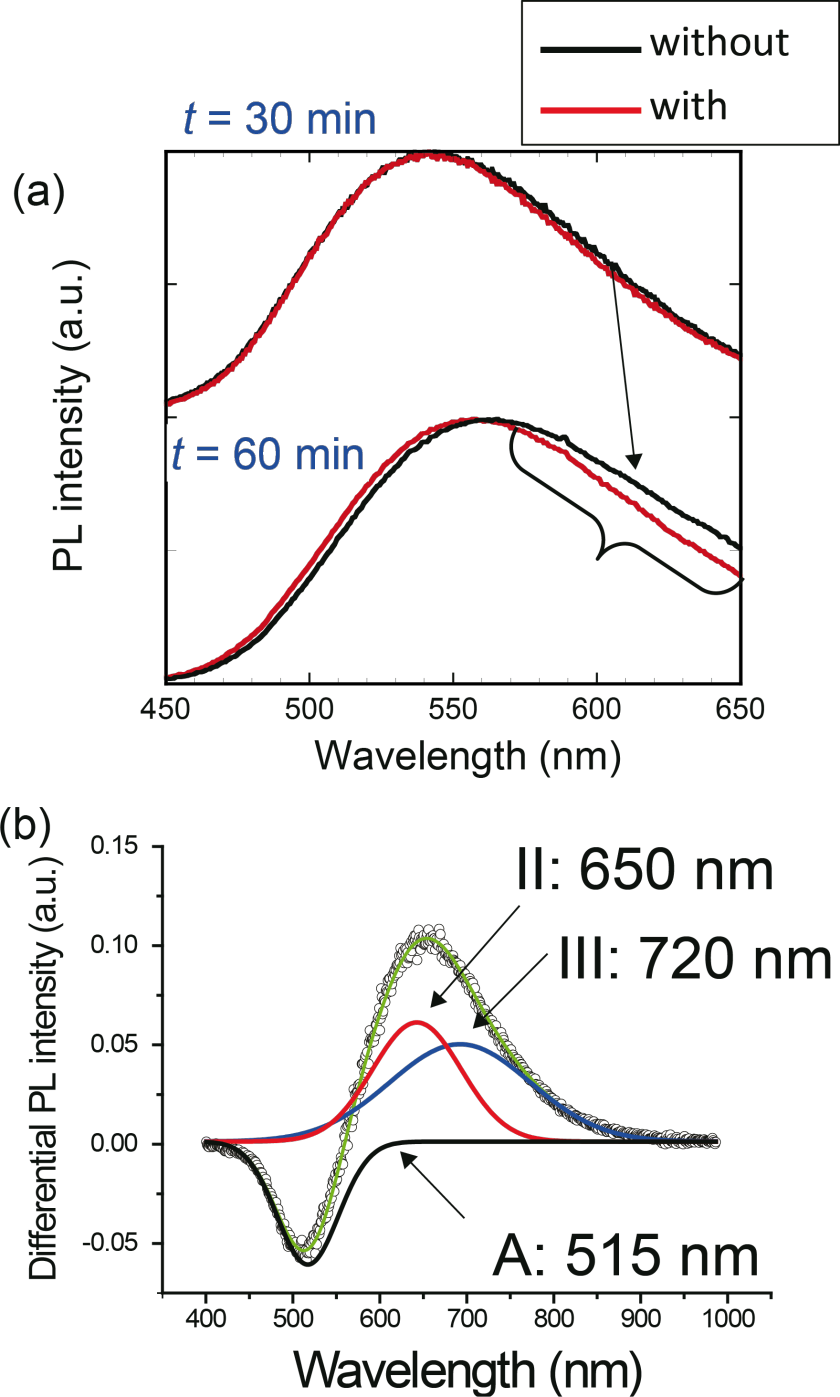
(a) PL spectra obtained with 593 nm light (λ_1_) as functions of the growth time, *t*. Black solid lines: spectra of nanocrystals obtained without light irradiation, *PL*_without_. Red solid lines: spectra of nanocrystals obtained with light irradiation, *PL*_with_. (b) Differential PL spectra calculated from the spectra obtained (after *t* = 60 min) with irradiation (red line in (a)) and without irradiation (black line in (a)).

To realize selective etching of the defect levels, ZAIS nanocrystals were synthesized with 593 nm light (λ_1_) illumination (10 mW) during the heat treatment in step (2). Figure 3a shows the dependence of the PL spectra obtained with the 325 nm light excitation on the growth time, *t*. A large spectral change was observed at *t* = 60 min at emission wavelengths λ > 550 nm, which was confirmed by obtaining the differential of the PL spectra (open circles in Figure 3b). The differential PL spectrum (= *PL*_without_ − *PL*_with_) was fitted by using three curves: curve A with a peak wavelength of 515 nm, curve II with a peak wavelength of 650 nm, and curve III with a peak wavelength of 720 nm. The peak wavelengths of curves II and III corresponded to those in Figure 2c, thus indicating that the impurity sites were selectively etched away. Meanwhile, the spectral peak around 515 nm increased (i.e., the differential PL intensity decreased), whose wavelength corresponded to the main emission peak in Figure 2c. These results indicate that the decrease in impurity sites resulted in the decrease of the non-radiative energy dissipation, and resulted in the increase of the emission intensity of the band edge.

### Using the emission spectra to control the nanocrystal size

**Figure 4:**
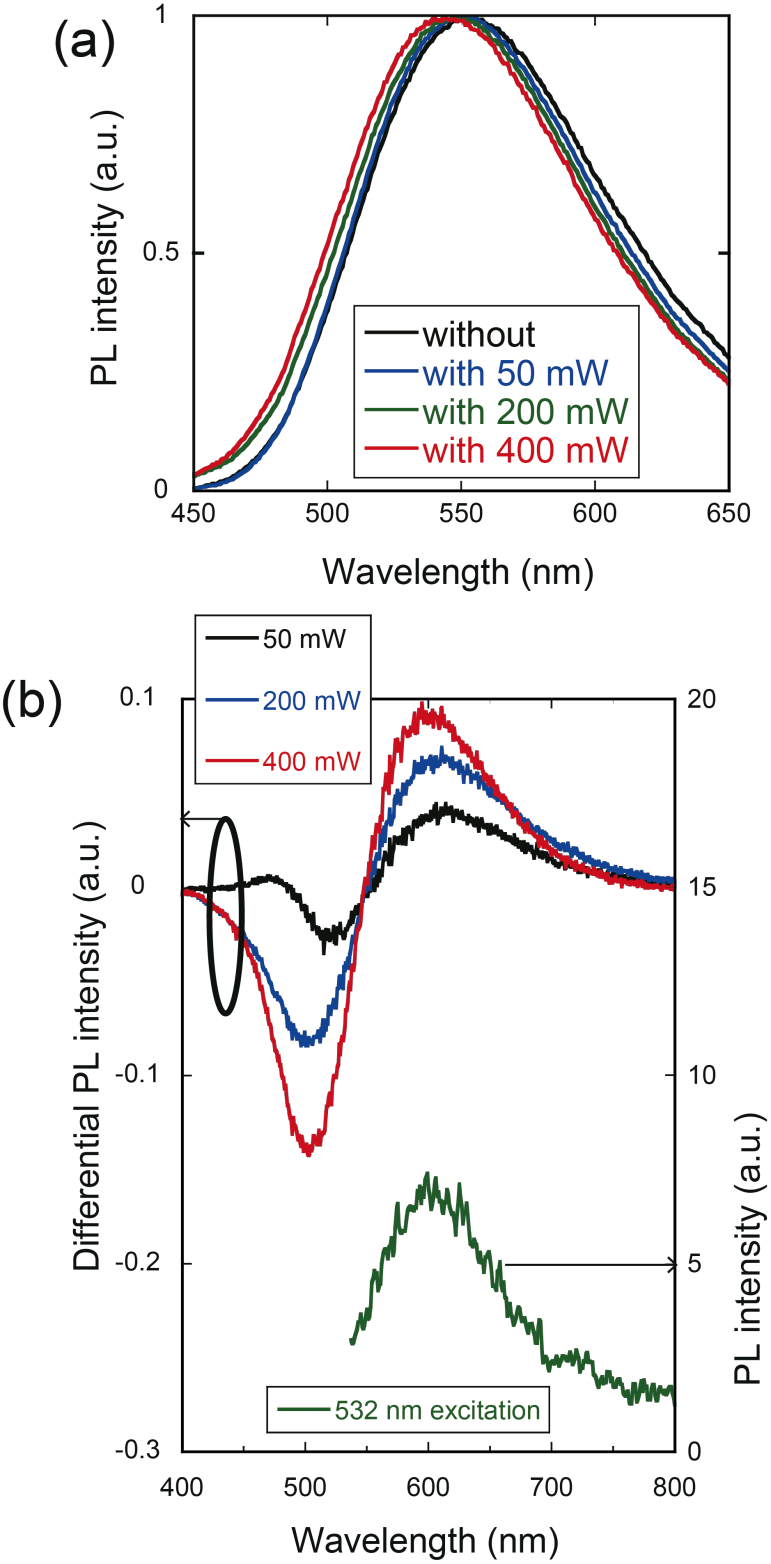
(a) PL spectra obtained with 532 nm light (λ_2_) and different levels of irradiation power. (b) (Left vertical axis) Differential PL spectra calculated from spectra obtained with and without irradiation. (Right vertical axis) PL spectrum of ZAIS nanocrystals grown without laser irradiation. The PL spectrum was obtained by using 532 nm light.

For the investigation of ways to control the size of ZAIS nanocrystals, we synthesized ZAIS nanocrystals with 532 nm irradiation (λ_2_) during the heat treatment in step (2). Figure 4a shows the PL spectra with different excitation power levels during the synthesis. From these spectra, the differential PL spectra (= *PL*_without_ − *PL*_with_) were obtained, as shown in Figure 4b. The positive value of the differential PL intensity at 600 nm indicates a decrease in the PL intensity of ZAIS nanocrystals synthesized with laser irradiation. Because the peak wavelength (600 nm) corresponded to the peak PL wavelength for ZAIS nanocrystals synthesized without laser irradiation, using an excitation wavelength of 532 nm (solid green line in Figure 4b), the decrease in PL intensity of the nanocrystals synthesized with 532 nm light excitation should have originated from carrier excitation by the 532 nm light. In addition, the differential PL intensity at shorter wavelengths around 500 nm was decreased, which indicates that the number of ZAIS nanocrystals with smaller size was increased.

### Transmission electron microscopy analysis of crystal size and shape

**Figure 5:**
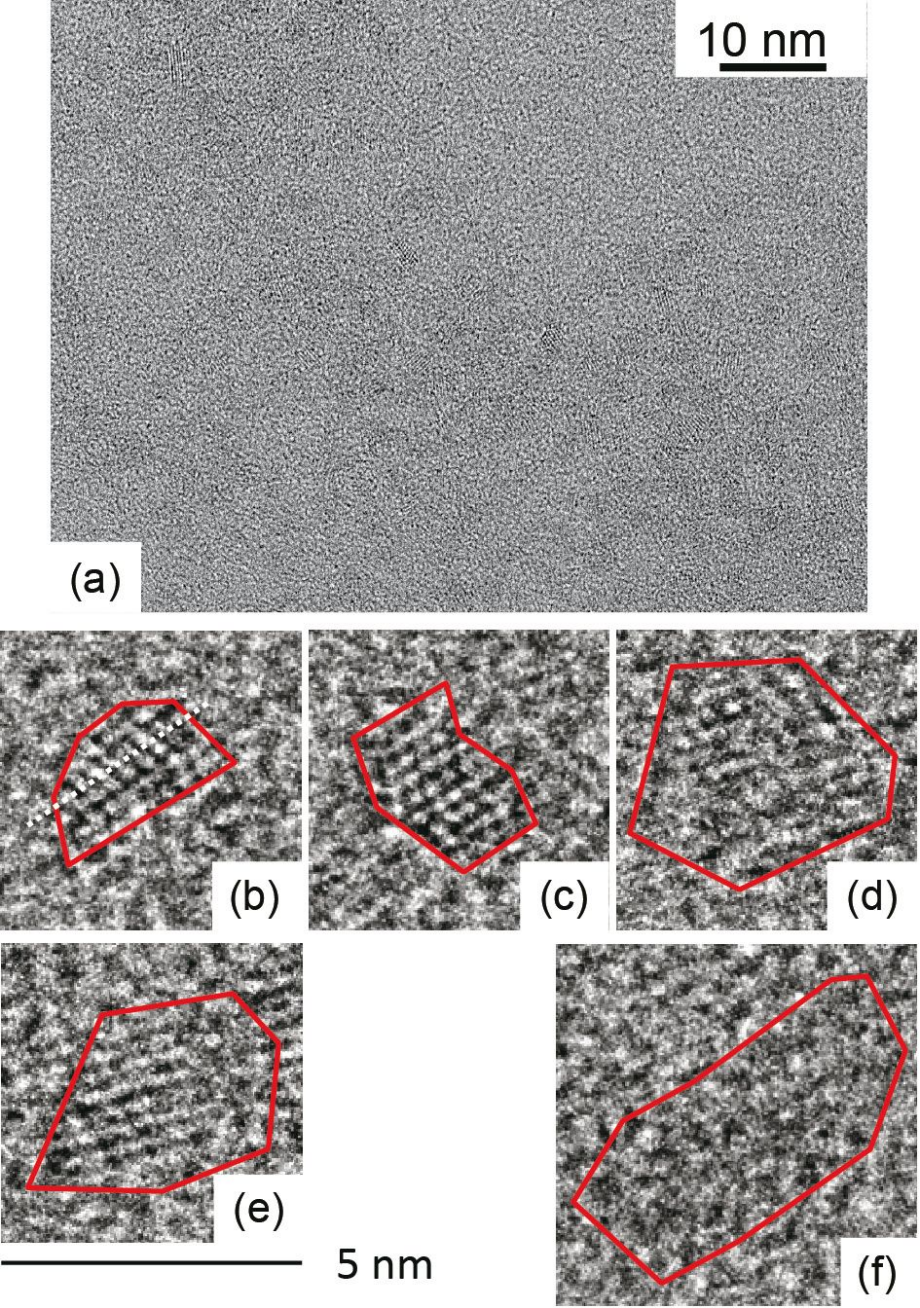
TEM images of ZAIS nanocrystals grown without irradiation, *t* = 60 min; (a) low (2,000,000×) and (b–f) high (4,000,000×) magnification.

**Figure 6:**
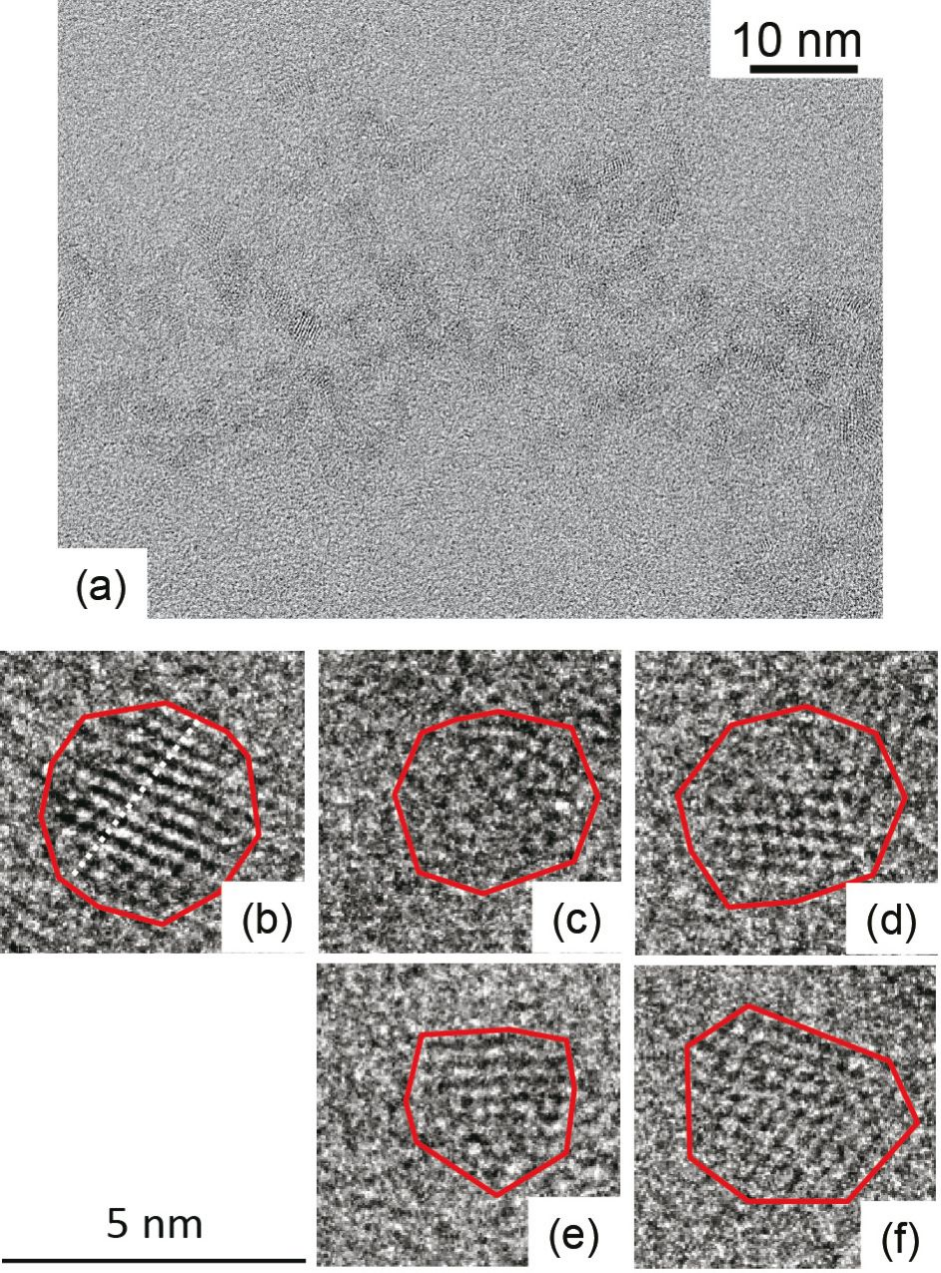
TEM images of ZAIS nanocrystals grown with 532 nm light (400 mW), *t* = 60 min; (a) low (2,000,000×) and (b–f) high (4,000,000×) magnification.

**Figure 7:**
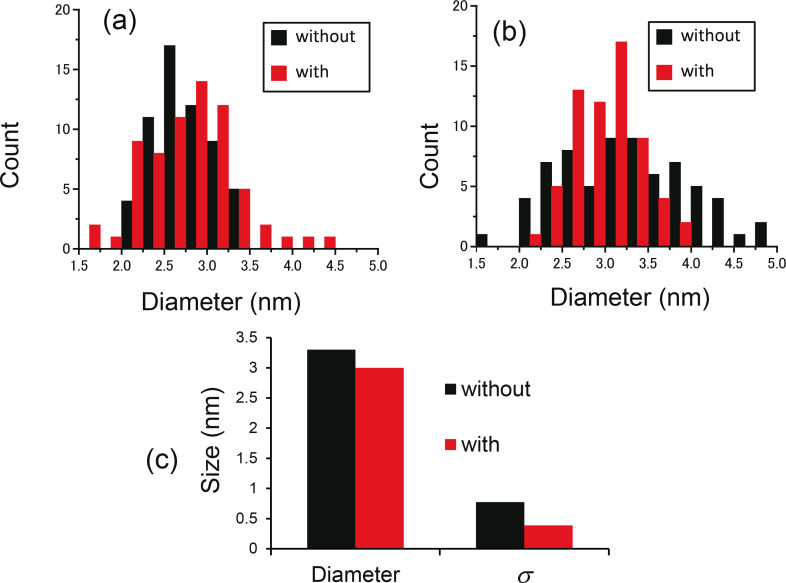
(a) Size distribution of among nanocrystals grown without and with 532 nm light (400 mW), *t* = 5 min. (b) Distribution of diameter among nanocrystals grown without and with 532 nm light (400 mW), *t* = 60 min. (c) Average diameter and σ.

Next, we evaluated the size distributions by using a transmission electron microscope (TEM, Hitachi H-9000NAR, acceleration voltage of 300 kV) to confirm the variations among the nanocrystals. Figure 5 and Figure 6 show the typical TEM images of synthesized ZAIS nanocrystals without and with 532 nm light (400 mW), obtained after *t* = 60 min, in which the red line indicates the outer shape of the nanocrystals. We evaluated the diameter by using high-magnification images (4,000,000×). We determined the edge of the nanocrystals by using the IMTool (Foundation for Promotion of Material Science and Technology of Japan). By taking the cross-sectional profiles, the edge of the nanocrystals is determined by the disappearance of the periodic contrast. Figure 7a and Figure 7b show the size distribution for growth times of *t* = 5 and 60 min, after using 532 nm light (400 mW) during the heat treatment. Although the actual shape of ZAIS nanocrystals was non-circular, the diameter was determined as the equivalent diameter of circles that would occupy the same amount of space as the ZAIS nanocrystals. Figure 7c shows the average diameter, 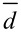, and the standard deviation, 
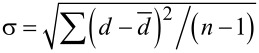
, of the diameter of ZAIS nanocrystals produced after *t* = 60 min. It indicates a decrease in diameter from 3.30 to 3.00 nm and a decrease in σ from 0.77 to 0.39 nm. These results support the postulates from Figure 4, namely the reduction of large ZAIS nanocrystals and the increase of smaller ZAIS nanocrystals by the irradiation of 532 nm light during synthesis.

**Figure 8:**
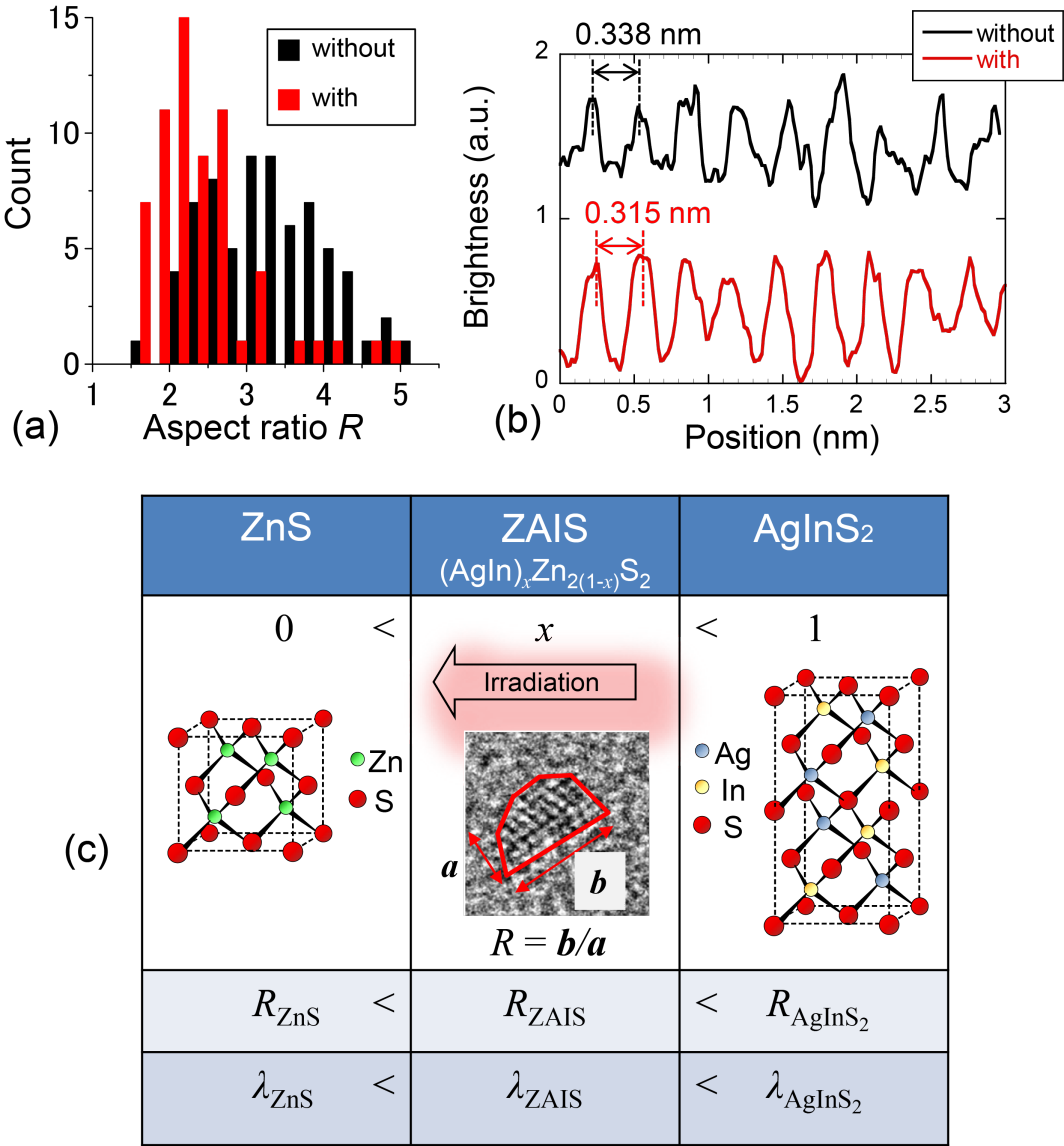
(a) Distribution of *R* among nanocrystals grown without and with 532 nm light (400 mW), for a growth time of *t* = 60 min. (b) Cross-sectional profiles of image brightness along the dashed white line in Figure 5b (without irradiation) and Figure 6b (with irradiation). (c) Schematic of the transition of the crystal structure of ZnS and AgInS_2_ depending on the composition.

In addition to examining the change in size distribution, we evaluated the aspect ratios of the nanocrystals by using TEM measurements. By using the TEM images (Figure 5 and Figure 6), we calculated the aspect ratio *R* (= *b*/*a*; *a*: shorter axis length, *b*: longer axis length, see Figure 8c). Figure 8a shows the distribution of values of *R* among ZAIS nanocrystals synthesized without and with irradiation of 532 nm light, at 400 mW for *t* = 60 min. The number of nanocrystals with larger *R* was decreased by introducing irradiation during the synthesis, and the value of the average *R* was decreased from 3.3 (without irradiation) to 2.4 (with irradiation). ZAIS consist of the solid solution (AgIn)*_x_*Zn_2(1−_*_x_*_)_S_2_. As *x* increases, it turns into AgInS_2_ with a tetragonal crystal structure; as *x* decreases, it becomes ZnS with a cubic crystal structure (see Figure 8c) [13]. Therefore, the observed decrease in the value of *R* should have originated from the reduction in Ag and In content.

To support the above postulate of the change in crystal structure by using photo-assisted synthesis, we evaluated the TEM images. The black and red curves in Figure 8b show the cross-sectional profiles of image brightness along the white dashed lines in the TEM images in Figure 5b and Figure 6b of ZAIS nanocrystals grown without and with irradiation (*t* = 60 min), respectively. From these results, the average separation of the peak-to-peak distances was determined to be 0.338 nm with a standard deviation σ of 0.019 nm for nanocrystals grown without irradiation and 0.315 nm with σ of 0.025 nm for nanocrystals grown with irradiation. These values are comparable to the reported values of 0.335 nm for AgInS_2_ nanocrystals [19] and 0.310 nm for ZnS nanocrystals [20]. These results also support the assertion that the observed decrease in *R* resulted from the reduction in Ag and In content. In other words, highly controlled nanocrystal size and uniform composition were realized by using photo-assisted synthesis.

## Conclusion

In conclusion, we attained precise size and composition control of ZAIS nanocrystals by introducing light irradiation during synthesis. The PL measurements and TEM analysis confirmed the reduction of the PL spectral width and a corresponding reduction of the size distribution of the nanocrystals. Furthermore, these results indicate that the synthesized ZAIS nanocrystals had a higher crystallinity; thus, higher energy-transmission efficiency can be expected for nanophotonic devices.
